# From ‘bridging specialties’ to ‘owning the patient’: rethinking accountability in Cardiovascular-Kidney-Metabolic syndrome care

**DOI:** 10.1093/ehjcvp/pvag057

**Published:** 2026-07-27

**Authors:** Lu Li, Qing Ni, Lan Lin

**Affiliations:** Department of Endocrinology, Guang’anmen Hospital, China Academy of Chinese Medical Sciences, No. 5 Beixiange, Guang'anmen Nei Street, Xicheng District, Beijing 100053, China; Department of Endocrinology, Guang’anmen Hospital, China Academy of Chinese Medical Sciences, No. 5 Beixiange, Guang'anmen Nei Street, Xicheng District, Beijing 100053, China; Department of Endocrinology, Guang’anmen Hospital, China Academy of Chinese Medical Sciences, No. 5 Beixiange, Guang'anmen Nei Street, Xicheng District, Beijing 100053, China

This commentary refers to ‘Implementing sodium-glucose co-transporter 2 inhibitors in cardiovascular-kidney-metabolic syndrome: a multidisciplinary expert perspective’ by A. Avogaro *et al*. https://doi.org/10.1093/ehjcvp/pvag020.

We commend Avogaro *et al*. for their timely and pragmatic expert consensus on overcoming the implementation gap of sodium–glucose co-transporter 2 inhibitors (SGLT2i) in cardiovascular–kidney–metabolic (CKM) syndrome.^1^ By meticulously cataloguing real-world barriers—from the Italian AMD registry showing only 41.9% SGLT2i uptake in type 2 diabetes to the BRING-UP 3 registry revealing 50.1% use in heart failure with preserved ejection fraction—the authors provide a compelling roadmap for cross-specialty integration. Their emphasis on simplified screening algorithms, shared diagnostic panels, and educational initiatives represents an essential step forward. However, we argue that the framework stops short of confronting a more fundamental structural question: who bears ultimate accountability for the holistic management of the patient with CKM syndrome?

The proposed solutions—bridging specialties through shared tools and communication—implicitly assume that existing specialty silos can be adequately connected. Yet the CKM paradigm, by its very nature, defies siloed ownership. Cardiologists focus on ventricular function, nephrologists on glomerular filtration, and endocrinologists on glycaemic control; each practises excellently within their domain, yet no single specialty is explicitly mandated or incentivized to orchestrate the continuum of care.^2^ This is not a failure of individual clinicians but a structural gap in accountability.

The consequences are twofold. First, therapeutic inertia becomes institutionalized when SGLT2i initiation depends on which specialist the patient happens to see rather than on clinical need. Second, cross-specialty coordination lacks sustainable incentives within performance metrics that remain specialty specific—a cardiologist is evaluated on left ventricular ejection fraction improvement, not on whether appropriate organ-protective therapy has been considered according to the patient’s CKM phenotype. Notably, this pattern mirrors the historical underuse of renin–angiotensin–aldosterone system inhibitors, confirming that evidence and guidelines alone, without systemic accountability redesign, cannot overcome therapeutic inertia.^3^

We propose three structural reforms to complement the authors’ framework. First, establish a ‘CKM-attending physician’ role—a cardiologist, nephrologist, or endocrinologist with certified training in integrated CKM management—who holds primary accountability for the patient’s longitudinal care, including the authority to initiate and titrate SGLT2i and other organ-protective therapies across traditional domain boundaries. This role differs fundamentally from a multidisciplinary team convener; it is a designated decision-maker with continuity of responsibility. Second, incorporate the rational use of SGLT2i into cross-specialty quality evaluation frameworks—the core objective is not to mechanically pursue prescription rates but to systematically assess whether SGLT2i and other organ-protective therapies have been duly considered and discussed—with clinical reasoning for initiation or deferral clearly documented—in patients with clear CKM indications. Evaluation should be grounded in ‘indication recognition rate’ and ‘decision documentation completeness’ rather than prescription rates *per se*, thereby making cross-specialty prescribing appropriateness measurable and traceable, while avoiding context-blind mandatory prescribing. Third, de-specialize prescribing authority by enabling appropriately trained generalists and specialists across disciplines to initiate SGLT2i, supported by standardized safety protocols—including the expected reversible estimated glomerular filtration rate dip and sick-day rules—as the authors have thoughtfully provided in their Table 3.

Avogaro *et al*. have laid a vital foundation. The next frontier is not merely building bridges between specialties but ensuring that every patient with CKM syndrome has a clinician who is accountable for their whole-person trajectory-transforming CKM from a conceptual framework into a deliverable care system centred on patient needs and guided by the principle of rational pharmacotherapy.^4^

## Data Availability

No new data were generated or analyzed in support of this research. All data referenced in this review are from previously published studies and are cited accordingly.
